# Thermally generated spin current in the topological insulator Bi_2_Se_3_

**DOI:** 10.1126/sciadv.adi4540

**Published:** 2023-12-13

**Authors:** Rakshit Jain, Max Stanley, Arnab Bose, Anthony R. Richardella, Xiyue S. Zhang, Timothy Pillsbury, David A. Muller, Nitin Samarth, Daniel C. Ralph

**Affiliations:** ^1^ Department of Physics, Cornell University, Ithaca, NY 14853, USA.; ^2^ School of Applied and Engineering Physics, Cornell University, Ithaca, NY 14853, USA.; ^3^ Department of Physics and Materials Research Institute, Pennsylvania State University, University Park, PA 16802, USA.; ^4^ Kavli Institute at Cornell for Nanoscale Science, Ithaca, NY 14853, USA.

## Abstract

We present measurements of thermally generated transverse spin currents in the topological insulator Bi_2_Se_3_, thereby completing measurements of interconversions among the full triad of thermal gradients, charge currents, and spin currents. We accomplish this by comparing the spin Nernst magneto-thermopower to the spin Hall magnetoresistance for bilayers of Bi_2_Se_3_/CoFeB. We find that Bi_2_Se_3_ does generate substantial thermally driven spin currents. A lower bound for the ratio of spin current density to thermal gradient is $\frac{J_{s}}{\nabla_{x}T}$ = (4.9 ± 0.9) × 10^6^ $\left(\frac{\hbar }{2e}\right)\frac{A\;m^{-2}}{K\;\text{μ}m^{-1}}$, and a lower bound for the magnitude of the spin Nernst ratio is −0.61 ± 0.11. The spin Nernst ratio for Bi_2_Se_3_ is the largest among all materials measured to date, two to three times larger compared to previous measurements for the heavy metals Pt and W. Strong thermally generated spin currents in Bi_2_Se_3_ can be understood via Mott relations to be due to an overall large spin Hall conductivity and its dependence on electron energy.

## Teaser

Topological insulator Bi2Se3 can transduce thermal gradients to transverse spin currents efficiently.

## INTRODUCTION

By taking advantage of the electron’s spin as well as its charge, the field of spin caloritronics has provided strategies for energy harvesting from thermal gradients, for thermal management within electronics, and for magnetic manipulation (*1*–*3*). However, the field has been limited by low efficiencies for interconversion between thermal gradients and spin currents within the materials studied to date. Here, we provide measurements of the efficiency of transduction from a thermal gradient to spin current density in a topological insulator. Topological insulators have already been demonstrated to provide very high efficiencies for interconversion between the other two legs of the triad of thermal gradients, charge currents, and spin currents. Topological insulators have achieved record efficiencies for transduction between charge current density and spin current density that are of interest for applications in spin-orbit torque manipulation of magnetic devices (*4*–*11*), and also highly efficient transduction of thermal gradients to electric field with potential for thermoelectric applications (*12*–*14*). We find that the topological insulator Bi_2_Se_3_ also enables highly efficient transduction of thermal gradients to spin currents, with by far the largest spin Nernst ratio among materials measured to date. Understanding thermally generated spin currents in topological insulators is important for characterizing the effect of Joule heating on measurements of current-induced spin-orbit torques (*15*). If the thermal spin currents are sufficiently strong, they could in principle be put to use in generating useful torques (*16*, *17*). The Mott relation, which connects the spin Nernst effect to the spin Hall effect, provides insight into the origin of the large effect we measure and also suggests how even stronger thermal-gradient to spin-current conversion might be achieved by optimizing topological insulators.

## RESULTS

We measure thermally generated spin currents using the same physics by which electrically generated spin currents give rise to the spin Hall magnetoresistance (SMR) effect (*18*). In the electrically generated case, an electric field *E* applied in the plane of a spin-source/ferromagnet bilayer gives rise to a vertically flowing spin current density *J_s_* via the spin Hall effect, with an efficiency characterized by the spin Hall ratio, θ*_SH_* (*19*): $J_{s}=\frac{\hbar }{2e}\frac{\theta_{SH}}{\rho_{SS}}E$, where ℏ is the reduced Planck constant, *e* is the magnitude of the electron charge, and ρ*_SS_* is the electrical resistivity of the spin-source material. The degree of reflection of this spin current at the magnetic interface depends on the orientation of the magnetization $\hat{m}$ in the magnetic layer. The reflected spin current produces a voltage signal by the inverse spin Hall effect, causing the resistance of the bilayer to depend on the magnetization angle (*18*, *20*, *21*): $\text{Δ}R(\hat{m})=(1-m_{y}^{2})\text{Δ}R_{SMR}$. Here, *m_y_* is the component of the magnetization unit vector that is in-plane and perpendicular to the electric field. This resistance change corresponds to a voltage signal amplitude

$$\text{Δ}V=\text{Δ}R_{SMR}I=\frac{\text{Δ}R_{SMR}}{R}El=\frac{2e}{\hbar }\frac{\text{Δ}R_{SMR}}{R}\frac{\rho_{SS}}{\theta_{SH}}lJ_{s}$$ (1)

where *I* is the total current through the bilayer, *R* is the total resistance of the bilayer, and *l* is the sample length. Our analysis will assume that a thermally generated spin current produces the same voltage signal as an electrically generated spin current (i.e., that Eq. 1 holds for both cases with the same experimentally measured value of Δ*R_SMR_*/*R*).

An in-plane thermal gradient ∇*_x_T* can similarly give rise to a vertically flowing spin current in a spin-source layer via the spin Nernst effect (*22*, *23*). Upon reflection of this spin current from a magnetic interface and then the action of the inverse spin Hall effect, this results in a voltage signal parallel to the thermal gradient that depends on the magnetization angle, in direct analogy to the SMR. For experiments measured with an open-circuit electrical geometry (i.e., no net longitudinal charge flow in the bilayer), we will define the efficiency of spin current generation by the spin Nernst effect in terms of a spin Nernst parameter θ*_SN_*

$$J_{s}=-\frac{\hbar }{2e}\frac{\theta_{SN}}{\rho_{SS}}S_{SS}\nabla_{x}T$$ (2)

Here, *S_SS_* is the Seebeck coefficient of the spin-source layer and ∇*_x_T* is the in-plane thermal gradient. The thermally generated voltage takes the form (*22*, *24*, *25*)

$$V_{th}^{x}=-l\nabla_{x}T[S+S_{1}+(1-m_{y}^{2})S_{SNT}]$$ (3)

where *S_SNT_* is the coefficient of the spin Nernst magneto-thermopower, *S*_1_ is a magnetization-independent offset arising from the spin Nernst effect and inverse spin Hall effect, and *S* denotes the total effective Seebeck coefficient of the bilayer given by $S=\frac{\rho_{SS}S_{FM}t_{FM}+\rho_{FM}S_{SS}t_{SS}}{\rho_{SS}t_{FM}+\rho_{FM}t_{SS}}\approx \chi S_{SS}$. This approximation holds when the spin-source layer is a topological insulator such as Bi_2_Se_3_ for which the Seebeck coefficient is much larger than for the ferromagnetic layer (*S_SS_* ≫ *S_FM_*), and we define a current shunting ratio χ = ρ*_FM_t_SS_*/(ρ*_FM_t_SS_* + ρ*_SS_t_FM_*), where ρ*_FM_* is the resistivity of the ferromagnet and *t_FM_* and *t_SS_* are the thicknesses of the two layers. As long as thermally generated and electrically generated spin currents are transduced to voltage the same way, we can combine Eqs. 1 to 3 to obtain

$$\frac{J_{s}}{\nabla_{x}T}=-S_{SNT}\frac{R}{\text{Δ}R_{SMR}}\frac{\hbar }{2e}\frac{\theta_{SH}}{\rho_{SS}}$$ (4)

$$\theta_{SN}=\theta_{SH}\frac{S_{SNT}}{S_{SS}}\frac{R}{\text{Δ}R_{SMR}}$$ (5)

These are the two equations we will use to evaluate the thermally generated spin current and the spin Nernst ratio θ*_SN_*.

For an open-circuit measurement, θ*_SN_* will have contributions from both spin current generated directly by a thermal gradient and spin current generated by an electric field that is also present because of the Seebeck effect. It is therefore also of interest to separate these effects and define a spin current that would be generated by a thermal gradient alone, in the absence of any electric field (*26*, *27*), i.e., to define a “bare” spin Nernst ratio $\theta_{SH}^{0}$ such that

$$J_{s}\equiv -\frac{\hbar }{2e}\theta_{SN}\frac{S_{SS}}{\rho_{SS}}\nabla_{x}T=\frac{\hbar }{2e}\frac{\theta_{SH}}{\rho_{SS}}E-\frac{\hbar }{2e}\frac{\theta_{SN}^{0}}{\rho_{SS}}S_{SS}\nabla_{x}T$$ (6)

$$=\frac{\hbar }{2e}(\chi \theta_{SH}-\theta_{SN}^{0})\frac{S_{SS}}{\rho_{SS}}\nabla_{x}T$$ (7)

Therefore, we can calculate

$$\theta_{SN}^{0}=\theta_{SN}+\chi \theta_{SH}$$ (8)

In our experimental geometry, a small vertical thermal gradient ∇*_z_T* can also be present when we apply an in-plane thermal gradient. This will produce additional background signals due to the ordinary Nernst effect (ONE), the spin Seebeck effect (SSE) + inverse spin Hall effect, and the anomalous Nernst effect (ANE):

$$V_{th}^{z}=V_{ONE}\nabla_{z}TB_{y}+V_{SSE}\nabla_{z}Tm_{y}+V_{ANE}\nabla_{z}Tm_{z}$$ (9)

These signals will be distinguished from the voltages arising from an in-plane thermal gradient based on the different dependences on the magnetization orientation $\hat{m}$.

**Fig. 1.**
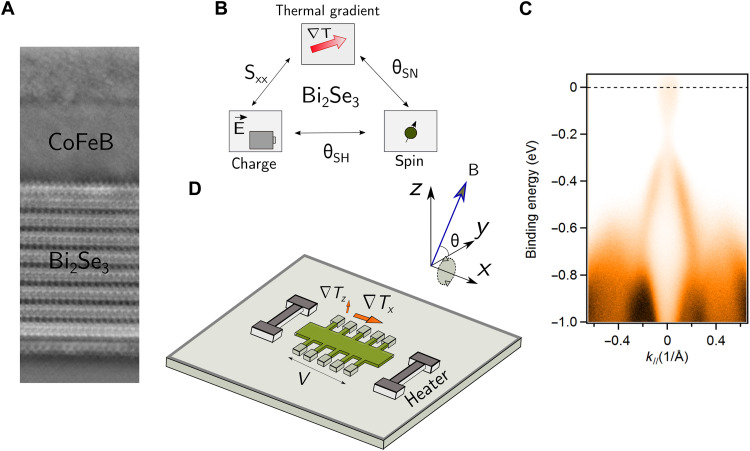
Sample geometry and photoemission spectrum of Bi_2_Se_3_. (**A**) Cross-sectional scanning transmission electron microscope image showing a sharp interface between Bi_2_Se_3_ and CoFeB. Bi_2_Se_3_ is crystalline, whereas CoFeB is amorphous. (**B**) Bi_2_Se_3_ excels at the three figures of merit associated with interconversion between spin, charge, and the thermal gradient. (**C**) ARPES measurements of the band structure of the top surface state of a single layer of Bi_2_Se_3_ of thickness 8 nm. The Fermi level is shown as the dotted line. *k*_ǁ_ denotes the Γ − *M* direction, and the spectrum is obtained at room temperature. (**D**) Sample geometry for the spin Nernst magneto-thermopower experiments.

We analyze bilayers of Bi_2_Se_3_ (8 nm)/Co_20_Fe_60_B_20_ (CoFeB) (5 nm). The 8-nm thickness of Bi_2_Se_3_ was chosen to ensure negligible hybridization between states on the two surfaces (*28*, *29*). The Bi_2_Se_3_ thin films were grown by molecular beam epitaxy (MBE) and initially capped with 20 nm of Se to protect them from air exposure while transporting them to a separate system for the deposition of CoFeB. Details about the MBE growth can be found in Materials and Methods. High-quality growth of Bi_2_Se_3_ is confirmed by atomic force microscopy as well as x-ray diffraction measurements (see fig. S1). The existence of a surface state on the Bi_2_Se_3_ thin films (with no Se cap or CoFeB overlayer) was also confirmed using angle-resolved photoemission spectroscopy (ARPES), which measured a Dirac-like dispersion as shown in Fig. 1C. As is evident from the position of the Fermi level in Fig. 1C, the Bi_2_Se_3_ layer is electron-doped before the Se capping. This is consistent with previous studies that have identified the cause of the doping to be Se vacancies (*13*). After transfer to the separate vacuum system, we heated the Bi_2_Se_3_/Se samples to a heater thermocouple temperature of 285°C for 3.5 hours to remove the Se cap. We then deposited CoFeB by dc magnetron sputtering followed by a 1.2-nm protective layer of Ta, which forms TaO*_x_* upon air exposure. Cross-sectional scanning transmission electron microscopy (Fig. 1A) shows that the bilayers have a sharp interface with no visible oxidation at the interface.

**Fig. 2.**
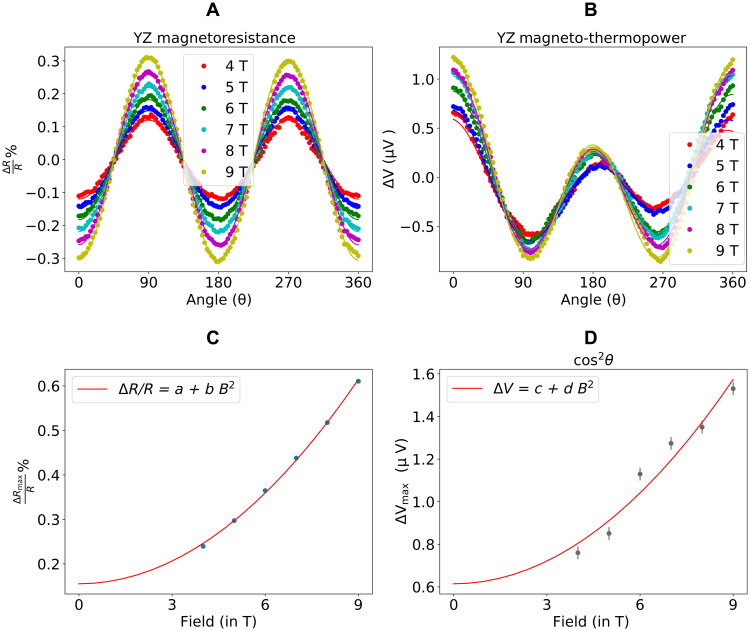
Electrical and thermal generation of spin currents. (**A**) Magnetoresistance percentage ratio ($\frac{\nabla R}{R}\times 100$) as a function of the magnetic field angle and magnitude for bilayers of Bi_2_Se_3_ (8 nm)/CoFeB (5 nm) at room temperature, for magnetic field rotated in the *YZ* plane. The four-point device resistance *R* = 2.137 kilohms. (**B**) *YZ* magneto-thermopower voltage as a function of the magnetic field angle and magnitude for the same sample. The heater power used for these sweeps was fixed at 442 mW (equivalent to a temperature drop of 6.8 K along the *l* = 1.8 mm length of the device). (**C**) Amplitude of the *YZ* magnetoresistance as a function of magnetic field magnitude, with a fit to the form 100 × (*a* + *bB*^2^). The fit yields *a* = 0.155 ± 0.05 and *b* = 0.0057 ± 0.0001 T^−2^. (**D**) Field dependence of the cos^2^θ part of *YZ* magneto-thermopower with a fit to the form *c* + *dB*^2^. The fit yields *c* = 0.61 ± 0.07 μV and *d* = 0.012 ± 0.001 μV/T ^2^.

We measure the spin current that is generated both electrically and thermally. As a first step, we measure the SMR of the bilayer. We use optical lithography to pattern a Hall bar sample geometry with nine pairs of Hall contacts (only five are depicted in Fig. 1D) and make a four-point measurement of the longitudinal resistance while rotating a magnetic field with fixed magnitude in the *YZ* plane as defined by the diagram in Fig. 1D. Since the magnetization is always perpendicular to the current flow for this orientation of field sweep, this provides a measurement of the SMR without contamination by the anisotropic magnetoresistance of the magnetic layer. Figure 2A shows the magnetoresistance data for magnetic field magnitudes of 4 to 9 T, large enough compared to the magnetic anisotropy (1.6 T) that to a good approximation the magnetization is saturated along the field direction. The data fit well to the angular dependence Δ*R*/*R* = Δ*R*_max_(1 − *m_y_*^2^) = Δ*R*_max_cos^2^θ. The amplitude of the magnetoresistance for different magnitudes of magnetic field is plotted in Fig. 2C. We find that the amplitude is well described by the dependence 100 × Δ*R*_max_/*R* = *a* + *bB*^2^, where *a* and *b* are constants.

In measurements of separate samples, we find that the *YZ* magnetoresistance of both an individual Bi_2_Se_3_ layer (fig. S5A) and an individual CoFeB layer (fig. S5C) also have cos^2^θ dependences. For the individual Bi_2_Se_3_ layer, the amplitude of this signal is purely quadratic in *B* with negligible zero-field offset (fig. S5B). For the full bilayer, we therefore identify the contribution that is quadratic in magnetic field (Fig. 2C) with the magnetoresistance of the topological insulator and the field-independent component as due primarily to the SMR. The *YZ* magnetoresistance of an individual CoFeB layer is weak, 100 × Δ*R*/*R* = 0.034 ± 0.003 (fig. S5D), but this is still about one-fifth the value for the full bilayer so that we take it into account as a small correction to the primary signal (see section S5). Assuming that the magnetoresistance of the CoFeB layer and the SMR contribute in parallel to the sample conductance, we estimate for the Bi_2_Se_3_/CoFeB bilayer that 100 × Δ*R*_SMR_/*R* = 0.126 ± 0.008.

We determine spin Nernst magneto-thermopower *S_SNT_* by measuring the thermal analog of *YZ* magnetoresistance, which, henceforth, we will refer to as the *YZ* magneto-thermopower. We use the experimental procedure similar to (*30*). We create a thermal gradient along the bilayer Hall bar using a lithographically defined Pt heater adjacent to one end of the Hall bar and measure the thermally induced longitudinal voltage between the fourth and last Hall contacts of the bilayer sample. Our samples contain two heaters, one near each end of the Hall bar, so that we can apply in-plane thermal gradients of either sign. For each given heater power, we determine the temperature drop along the sample by measuring the local temperature at the position of the probed pairs of Hall contacts. This is done by measuring the change in the two-point resistance for each pair of Hall contacts and comparing to measurements of resistance versus temperature when an external heater is used to heat the full sample chip uniformly.

**Fig. 3.**
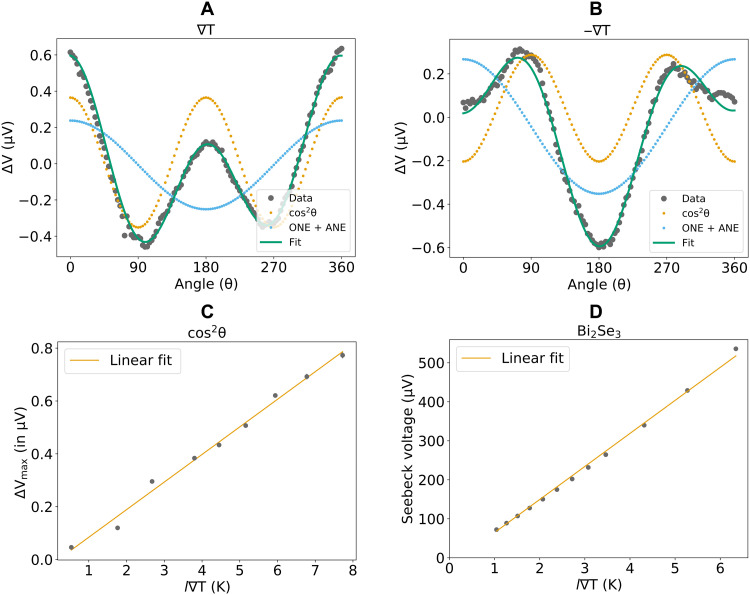
Dependence of the thermally generated voltages on heater polarity and power. (**A** and **B**) *YZ* magneto-thermopower for a field magnitude of 4 T and a heater power of 335 mW (corresponding to a temperature drop of 5.16 ± 0.05 K across the device) for (A) one sign of in-plane temperature gradient and (B) the opposite in-plane thermal gradient. (**C**) Dependence of the cos^2^θ signal on the temperature difference across the device. (**D**) Seebeck voltage for an 8-nm Bi_2_Se_3_ layer as a function of the temperature difference across the device. The Seebeck coefficient relative to the Ti/Pt electrodes was determined to be −87 ± 1 μV/K, consistent with a previous study (*14*).

The measured *YZ* magneto-thermopower of the Bi_2_Se_3_ bilayer for magnetic field magnitudes from 4 to 9 T is shown in Fig. 2B. Figure 3 (A and B) shows a representative analysis of the dependence on magnetic field angle for two 4-T scans with opposite orientations of in-plane thermal gradients. We observe two distinct contributions to the dependence of the thermopower on magnetic field angle, ∝cosθ and ∝cos^2^θ. The cosθ contribution can be understood as due to the terms arising for an out-of-plane component of the thermal gradient (Eq. 9). As expected, this contribution retains the same sign when the direction of the in-plane thermal gradient is reversed (compare Fig. 3A to Fig. 3B).

**Fig. 4.**
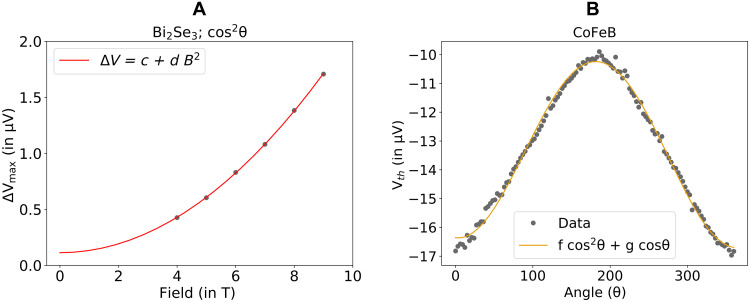
Control experiments that show negligible contributions to the spin Nernst magneto-thermopower from single layers of Bi_2_Se_3_ or CoFeB. (**A**) Field dependence of the cos^2^θ component of the *YZ* magneto-thermopower from an 8-nm Bi_2_Se_3_ film. The thermal difference was fixed at 2.17 ± 0.02 K. This is fit to the form *c* + *dB*^2^ with *c* = 113 ± 25 nV and *d* = 0.020 μV/T^2^. (The contribution in a bilayer after accounting for shunting would therefore be just $\frac{c\chi }{\text{Δ}T}$ = 8.3 ± 1.8 nV/K.) (**B**) Angle dependence of the *YZ* magneto-thermopower signal for a 5-nm CoFeB film at 5 T for the heater power of 318 mW. The fit is to the form *f*cos^2^θ + *g*cosθ, with *f* = 0.04 ± 0.02 μV (indicating a negligible spin Nernst effect) and *g* = −3.14 ± 0.02 μV.

The cos^2^θ component within the *YZ* magneto-thermopower of the bilayer is our primary focus; it changes sign upon reversal of the in-plane thermal gradient (Fig. 3, A and B) as expected for a Nernst effect. We can understand the dependence of this component on the magnitude of the applied magnetic field (Fig. 2D) by comparison to the contributions from the individual layers by themselves. Similar to the case of the *YZ* magnetoresistance, the *YZ* magneto-thermopower of a Bi_2_Se_3_ layer by itself has a cos^2^θ component whose amplitude is quadratic in *B* with at most a very small zero-field offset that would negligibly for a bilayer after accounting for shunting (Fig. 4A). This similarity between the magnetoresistance and magneto-thermopower is a consequence of the Mott relation (see section S6). The magneto-thermopower of the CoFeB layer by itself (Fig. 4B) contains only a cosθ dependence to measurement accuracy, corresponding to the anomalous Nernst voltage, with no cos^2^θ signal. For the full Bi_2_Se_3_/CoFeB bilayer, the amplitude of the magnetic field–dependent part of the cos^2^θ component of the *YZ* magneto-thermopower agrees quantitatively with the amplitude expected from the quadratic dependence of the single layer of Bi_2_Se_3_ after accounting for shunting (see section S4). Therefore, just as for the magnetoresistance, we fit the amplitude of the *YZ* magneto-thermopower for the bilayer to the form Δ*S* = *c* + *dB*^2^ with constants *c* and *d* (see Fig. 2D), and we identify the field magnitude–independent part as due to the spin Nernst effect. For the *l* = 1.8 mm device with a heater power of 442 mW (*l*∇T = 6.8 ± 0.1 K), we find *S_SNT_* × *l*∇ *T* = 610 ± 70 nV. Figure 3C shows (as expected) that the cos^2^θ term of the *YZ* magneto-thermopower varies linearly with temperature difference across the device (i.e., for different heater powers). We have also repeated the full analysis with a second Bi_2_Se_3_/CoFeB bilayer with a slightly different CoFeB thickness (6 nm rather than 5 nm) with consistent results (fig. S7).

To convert our measurement of *S_SNT_* to the spin Nernst ratio θ*_SN_* using Eq. 5, we must also determine the absolute Seebeck coefficient *S_SS_* and the spin Hall ratio θ*_SH_* of the Bi_2_Se_3_ spin source. To measure the Seebeck coefficient of Bi_2_Se_3_, we grew a layer of Bi_2_Se_3_ (8 nm) capped with 20 nm of Se, removed the Se with the same process used for the Bi_2_Se_3_ samples, and then grew 1.2 nm of Ta, which oxidizes upon air exposure. The Seebeck coefficient was measured using a similar sample geometry as the spin Nernst magneto-thermopower (Fig. 1D), but in the absence of a magnetic field. We obtain a Seebeck coefficient for 8-nm Bi_2_Se_3_ relative to the Ti(5 nm)/Pt(70 nm) electrodes to be −87 $\frac{\mu V}{K}$ (Fig. 3D). The absolute Seebeck coefficient of the electrodes should be small [≈−1 $\frac{\mu V}{K}$ (*31*)], so we estimate that the absolute Seebeck coefficient of the 8-nm Bi_2_Se_3_ is *S_SS_* = −86 $\frac{\mu V}{K}$ with a few percent uncertainty. The measured Seebeck coefficient *S* of the full bilayer is quantitatively consistent using the measured value *S_SS_* for the isolated Bi_2_Se_3_ layer and the estimated shunting parameter χ of the bilayer (see section S4).

Given our measurements that *S_SNT_* = 90 ± 10 $\frac{nV}{K}$, *S_SS_* = −86 $\frac{\mu V}{K}$, and Δ*R_SMR_*/*R* = *a*/100 = (1.26 ± 0.08) × 10^−3^, Eq. 5 yields the ratio of the spin Nernst ratio to the spin Hall ratio to be θ*_SN_*/θ*_SH_* = −0.83 ± 0.15. By Eq. 8, the corresponding ratio for the bare spin Nernst ratio is θ*_SN_*^0^/θ*_SH_* = −0.67 ± 0.15. Values of order 1 for the ratios θ*_SN_*/θ*_SH_* and θ*_SN_*^0^/θ*_SH_* have also been measured for heavy metals (*22*, *24*, *25*, *30*).

To determine the values of *J_s_*/∇*_x_T*, θ*_SN_*, and θ*_SN_*^0^ by themselves, we must also know the spin Hall ratio θ*_SH_* for the Bi_2_Se_3_ layer. To estimate this quantity, we performed a second harmonic Hall analysis (*32*) for the Bi_2_Se_3_/CoFeB bilayer (see section S3). This analysis yields a spin torque efficiency ξ*_DL_* that is related to θ*_SH_* according to ξ*_DL_* = *T*_int_θ*_SH_*, where *T*_int_ ≤ 1 is an interfacial spin transparency factor. We find ξ*_DL_* = 0.73 ± 0.02, which therefore provides a lower bound for θ*_SH_*. We conclude that a lower bound for *J_s_*/∇*_x_T* is (4.9 ± 0.9) × 10^6^ (ℏ/2*e*) A m^−2^/K μm^−1^, that a lower bound for the magnitude of θ*_SN_* is approximately −0.61 ± 0.11, and that a lower bound for the magnitude of θ*_SN_*^0^ is −0.49 ± 0.09. Comparisons to previous measurements of heavy metals are provided in Table 1.

**Table 1.** Comparison of the different figures of merit for the bilayers studied in this work with the existing literature. *S_SS_* was measured in (*24*, *30*) and calculated in (*22*), while (*25*) referenced existing literature for W and Pt samples. Values for $\frac{J_{s}}{\nabla T}$ for previous studies are calculated using parameters in the table.

	Bi_2_Se_3_	W			Pt	
	This work	(*30*)	(*24*)	(*25*)	(*22*)	(*25*)
θ*_SN_*	−0.61 ± 0.11	–	0.2	0.32 ± 0.1	−0.2	−0.18 ± 0.06
$\frac{J_{s}}{\nabla T}$ (A m^−2^/K μm^−1^)	(4.9 ± 0.9) × 10^6^	–	1.84 × 10^6^	(2.6 ± 0.8) × 10^6^	1.21 × 10^6^	(6 ± 2) × 10^6^
$\frac{\theta_{SN}}{\theta_{SH}}$	−0.83 ± 0.15	−2.4 ± 0.6	−0.7	−1.53 ± 0.47	−0.6	−1.8 ± 0.6
ξ*_DL_*	0.73	–	−0.28	−0.21	0.11	0.1
*S_SS_* ($\frac{\mu V}{K}$)	−86.6	−4.5	−12	10	−2.6	−10
ρ*_SS_* (microhm · cm)	1064	204	130	125	43	30

## DISCUSSION

The Mott relations, which connect electrical and thermoelectric transport coefficients, provide insight about why the spin Nernst ratio can be large in topological insulators and how it might be further optimized. According to the Mott relations for a system with degenerate electron statistics, the thermally generated spin current can be related to the energy derivative of the spin Hall conductivity

$$\frac{J_{s}}{\nabla T}=-\frac{\hbar }{2e}\frac{\pi^{2}k_{B}^{2}T}{3e}\frac{\partial \sigma_{SH}}{\partial E}$$ (10)

so that the spin Nernst ratio is given by the relation

$$\theta_{SN}=-\frac{\frac{\partial \sigma_{SH}}{\partial E}}{\frac{\partial \sigma_{SS}}{\partial E}}$$ (11)

where σ*_SS_* = 1/ρ*_SS_* (see section S6). As a consequence, θ*_SN_* is enhanced, in general, by having a large overall scale for σ*_SH_* and a relatively small value for σ*_SS_*, both of which can be provided by topological insulators. More directly relevant than these overall scales, however, are the energy derivatives ∂σ*_SH_*/∂*E* and ∂σ*_SS_*/∂*E*. This dependence on energy derivatives can explain why, although the spin Nernst ratio in Bi_2_Se_3_ is enhanced relative to the heavy metals, the degree of enhancement we measure is less than for the corresponding spin Hall ratios. Moreover, further optimization of thermally generated spin currents might be achieved by tuning the Fermi level in the topological insulator by doping to maximize ∂σ*_SH_*/∂*E* and minimize ∂σ*_SS_*/∂*E*. The experiments we report have so far been performed at a fixed stoichiometry for Bi_2_Se_3_, but previous experiments have measured strong variations of the spin Hall effect with Fermi level by varying elemental compositions (*6*, *33*, *34*), and such variations are also predicted by theory (*35*).

Last, we address an important question regarding these enhancements in charge/spin and thermal-gradient/spin conversion efficiency: Do the topological surface states play a role? The short spin diffusion length in Bi_2_Se_3_ (*8*) suggests that states at the surface must be involved. However, we note that there is currently no known experimental technique that can ascertain whether the topological nature of the surface states shown in Fig. 1C survives after the deposition of a metallic ferromagnetic overlayer. First-principles calculations indicate that the surface states at such interfaces are likely to be complicated (*36*, *37*). Nonetheless, recent studies have shown that the density of Berry curvature in the bulk band structure of topological insulators such as Bi_2_Se_3_ leads to a large spin Hall conductivity. Bulk-surface correspondence then implies that efficient spin-charge interconversion will also occur via surface states (*35*).

In summary, by comparing measurements of the spin Nernst magneto-thermopower in Bi_2_Se_3_/CoFeB bilayers to the SMR, we find a lower bound for the magnitude of the spin Nernst ratio for Bi_2_Se_3_ of −0.62 ± 0.11, roughly three times greater than that of previously measured values for Pt and two to three times greater than that of W. Moreover, the net spin current generated per unit thermal gradient *J_s_*/Δ*T_x_* is higher in Bi_2_Se_3_ than for W and of a similar magnitude as Pt despite the higher resistivity of Bi_2_Se_3_. The Mott relation connecting the spin Nernst effect and the spin Hall effect suggests an avenue by which thermally generated spin currents in topological insulators might be optimized even further, by taking advantage of both the large overall scale of the spin Hall conductivity in topological insulators and the strong dependence of the spin Hall conductivity on electron energy.

## MATERIALS AND METHODS

### Thin-film growth

The Bi_2_Se_3_ films were grown by MBE in a ScientaOmicron EVO50 system. Sapphire (0001) substrates were initially outgassed in vacuum at roughly 600°C and were then cooled to a growth temperature of 220°C, as measured by a pyrometer with an emissivity of 0.7. Bismuth (99.999%) and selenium (99.999%) from Alfa Aesar were coevaporated with a flux ratio of 1:10 until a thickness of 8 nm was achieved. The Bi_2_Se_3_ films were then capped with 20 nm of Se. The Se capping layers are amorphous and are deposited with a manipulator (substrate) temperature of 10°C, with the Se crucible at 210°C and the Se cracker at 350°C. The film thicknesses were calibrated using both atomic force microscopy and x-ray reflectivity. After the samples were transferred through air to a separate vacuum system for deposition of the magnetic layer, the films are heated to 285°C for 3.5 hours to remove the selenium cap. Sputter deposition (30 W in an argon pressure of 2 mtorr) was then used to grow layers of CoFeB (5 nm) and Ta (1.2 nm). The purpose of the Ta cap is to protect the CoFeB layer from oxidizing.

### Device fabrication and layer resistivities

Hall bar devices were used for measuring both the magnetoresistance and magneto-thermopower of the Bi_2_Se_3_/CoFeB devices. These were fabricated using three-step photolithography. First, the Hall bars themselves (2500 μm long by 200 μm wide with nine pairs of Hall contacts; only five are depicted in Fig. 1D) were defined and etched using an Ar-ion plasma. Then, heaters positioned 15 μm beyond each end of the Hall bar were defined using optical lithography followed by sputtering of Ti (5 nm)/Pt (≈24 nm). In the final step, electrical contacts were defined using lithography followed by sputtering of Ti (5 nm)/Pt (≈70 nm) electrodes. The masks used for fabrication can be found with the additional files on Zenodo (*38*). Control samples consisting of single layers of Bi_2_Se_3_ and CoFeB were fabricated similarly. We measured the magneto-thermopower between the fourth and the ninth Hall contacts away from a heater to reduce the unintentional out-of-plane thermal gradient present in the devices (*30*).

The four-point resistivity of an individual 8-nm layer of Bi_2_Se_3_ was measured to be 1064 microhm·cm, and the resistivity of an individual 5-nm layer of CoFeB was measured to be 128 microhm·cm. The overall resistance of a 2200-μm-long, 200-μm-wide channel of the Bi_2_Se_3_ bilayer was 2329 ohms. This compares well to the value expected (=2360 ohms) from adding the conductances of the individual layers.

### Characterization of the Bi_2_Se_3_ layers by ARPES

After growth, the band structure of the Bi_2_Se_3_ films (with no capping layer) was measured using in situ ARPES, performed with the 21.2-eV helium I α spectral line from a helium plasma lamp and a ScientaOmicron DA 30L analyzer with 6-meV energy resolution (see Fig. 1C).

### Details of the electrical and thermal measurements

The magnetoresistance and magneto-thermopower measurements were carried out in Quantum Design Physical Properties Measurement System (PPMS) with a horizontal rotator. The PPMS was interfaced with a home-built breakout box and software for external control. The experiments were carried out with the samples at room temperature.

## Supplementary Materials

### This PDF file includes:

Supplementary Text Figs. S1 to S7 References
