# Supplementary material for: Thermally generated spin current in the topological insulator Bi2Se3
Source: Sci Adv. 2023 Dec 13;9(50):eadi4540. doi: 10.1126/sciadv.adi4540 (PMC10848729; doi:10.1126/sciadv.adi4540)
Supplement: Supplementary file 1 — Supplementary Text Figs. S1 to S7 References [file sciadv.adi4540_sm.pdf]

Science Advances
AAAS

# Supplementary Materials for

## Thermally generated spin current in the topological insulator $Bi_2Se_3$

Rakshit Jain *et al.*

Corresponding author: Rakshit Jain, rj372@cornell.edu; Daniel C. Ralph, dcr14@cornell.edu

*Sci. Adv.* **9**, eadi4540 (2023)
DOI: 10.1126/sciadv.adi4540

**This PDF file includes:**

Supplementary Text
Figs. S1 to S7
References

# I. Materials characterization of the $Bi_2Se_3$ layers

See Fig. S1.

# II. Thermal gradient calibration

The thermal gradient within the sample Hall bars produced by the on-chip heaters was calibrated using the temperature dependence of the sample resistivity, as shown in Fig. S3. The thermal difference was measured between two points A and B on the device (the same points between which the YZ magneto-thermopower is measured). This was done in the following steps:

- First, the changes in resistance for the pairs of side contacts at positions A and B were measured as a function of temperature. (Fig. S3(B)). These data were used to calibrate the linear temperature coefficient for points A, B; i.e., for each point to determine the linear temperature coefficient $\alpha$ in the expression: $100 \times \frac{\Delta R}{R} = (\alpha(T - T_0))$ where $T_0$ is the reference temperature (305 K for this calibration).
- Second, the change in resistance as a function of increasing heater power was measured for the pairs of side contacts at points A and B. Using the values of $\alpha$ noted in the first step, the temperature of points A and B was calculated (Fig. S3(C)), along with the thermal difference between these two points (Fig. S3(D)).

# III. Measurement of spin Hall torque efficiencies

To determine the spin Hall torque efficiency we performed second harmonic hall (SHH) measurements (*32, 39*) on $Bi_2Se_3$ (8 nm)/CoFeB (2.6 nm) bilayers. (We used a thinner CoFeB layer than for the magneto-themopower measurements in the main text in order to enhance the

torque signals. For these samples we estimate that the current shunting ratio is $\chi$ = 0.29.) The bilayers were etched into a Hall bar shape with width 6 μm and length 20 μm, with side contacts 5 μm wide. The device resistance is 1340 Ohms. We applied a low-frequency alternating current (1317 Hz in this experiment) of $I_{RMS}$ = 3 mA through the device and measured the Hall-voltage response at the 1st and 2nd harmonic frequencies (1317 Hz and 2634 Hz). These data are plotted in Fig. S4.

The first and second harmonic response in presence of in plane external field (H) can be fitted to the following expressions (we ignore in-plane anisotropy) (*32*)

$$V_{\Omega} = V_{PHE}\ \sin 2\phi + V_{AHE}\ \cos\theta \tag{12}$$

$$V_{2\Omega} = \frac{\Delta H_{FL} V_{PHE}}{H} \cos 2\phi \cos\phi + \left( \frac{\Delta H_{DL} V_{AHE}}{2(H + H_{\perp})} + V_{ANE} + V_{ONE} H \right) \cos\phi, \tag{13}$$

where $V_{PHE}$ denotes the planar Hall voltage, $V_{AHE}$ denotes the anomalous Hall voltage, $V_{ANE}$ denotes the anomalous Nernst voltage, $V_{ONE}$ denotes the ordinary Nernst voltage, and $H_{\perp}$ denotes the field required to saturate CoFeB in the out of plane direction. $\Delta H_{FL}$ denotes effective field corresponding to the field-like torque and $\Delta H_{DL}$ denotes the effective field corresponding to the damping-like torque. From the fits we obtain $\Delta H_{DL}$ to be 16.4 Oe and $\Delta H_{FL}$ to be 1.36 Oe. The contribution from the Oersted field ($\frac{\mu_0 J_e t_{BS}}{2}$) is estimated to be 0.97 Oe. These data can be converted into damping and field-like torque efficiencies given by

$$\xi_{DL,FL} = \frac{2e}{\hbar} \frac{\mu_0 M_s t_{CFB} \Delta H_{DL,FL}}{J_e} \tag{14}$$

where $J_e$ denotes the current density through the $Bi_2Se_3$ layer, $M_s$ denotes saturation magnetization which is measured to be 1.53/$\mu_0$ T using vibrating sample magnetometry, and $t_{CFB}$ denotes the thickness of the CoFeB magnetic layer. From this analysis we obtain $\xi_{DL}$ to be 0.73 $\pm$ 0.02 and $\xi_{FL}$ to be 0.02 $\pm$ 0.01.

One potential concern about performing second harmonic Hall measurements of the spin-orbit torque from a topological insulator is that there could be artifacts arising from non-linear

Hall contributions unrelated to the spin torque (*40*). However, this effect appears to be important primarily at low temperatures, and has been shown to be have negligible at temperatures above 200 K (*41*). The measurements we present are limited to room temperature.

# IV. Comparison of Seebeck coefficients in bilayer vs single layer of $Bi_2Se_3$

From the background voltage of the measurement in Fig. 2(B), the Seebeck coefficient of the bilayer is determined to be $S = -13.27 \pm 0.25\ \frac{\mu V}{K}$. The resistivity of an individual 8 nm $Bi_2Se_3$ layer is 1064 μOhm cm and for an individual 5 nm CoFeB layer is 128 μOhm cm. We therefore estimate that the shunting coefficient of the bilayer is $\chi = 0.16$. After accounting for shunting, we estimate that for the $Bi_3Se_3$ layer in the absence of shunting $S_{SS} = S/\chi = -83 \pm 2$ $\frac{\mu V}{K}$. The close consistency with the value measured directly for the individual $Bi_2Se_3$ layer ($-86$ $\frac{\mu V}{K}$, see the main text) suggests that the thermoelectric properties of the $Bi_2Se_3$ in the bilayer are not strongly changed by interaction with the CoFeB layer.

# V. YZ Magnetoresistance in single layers of $Bi_2Se_3$ and CoFeB

In order to test for any contribution to the spin Hall magnetoresistance of the bilayer arising from the individual layers of $Bi_2Se_3$ and CoFeB, we performed YZ magneto-resistance measurements on single layers of $Bi_2Se_3$ (8 nm) and CoFeB (5 nm), both capped with 2 nm of Ta which oxidizes upon exposure to air. The YZ magnetoresistance of $Bi_2Se_3$ is plotted in Fig. S5(A) and the field dependence of the $\cos^2\theta$ fits is plotted in Fig. S5(B). A fit of the data in Fig. S5(B) to the form $a + bB^2$ with fitting parameters $a$ and $b$ indicates a substantial contribution $\propto B^2$, but the zero-field extrapolation is just $a = 0.0020 \pm 0.0006$, which is negligible compared to the SMR signal 0.155 in the main text. We therefore observe a negligible spin Hall magnetoresistance signal for the single layer of $Bi_2Se_3$.

For CoFeB we carried out similar YZ magnetoresistance measurement, finding the angular dependence plotted in Fig. S5(C). A fit of the data in Fig. S5(D) to the form $a + bB^2$ with fitting parameters $a$ and $b$ yields $a = 0.034 \pm 0.003$ and $b = (4.4 \pm 0.6) \times 10^{-5}$ T$^{-2}$. The field-independent part important for this study ($= a$) is non-zero, but roughly 5 times smaller than the the SMR signal 0.155 in the main text. This non-zero value could arise from the asymmetry in our structure ($Al_2O_3$/CoFeB/$TaO_x$) (*42*). Similar signals have been reported in CoFeB/MgO samples in Fig. S4(B) of (*24*). If we assume that the CoFeB signal and the SMR contribute in parallel to the conductance of the bilayer and subtract off the CoFeB signal, our estimate for the SMR signal is $100 \times \Delta R_{SMR}/R = 0.126 \pm 0.08$.

We have also compared the magnetic-field dependences of the YZ magneto-thermopower and magnetoresistance between the $Bi_2Se_3$/CoFeB bilayer and the individual $Bi_2Se_3$ films capped with oxidized Ta. For the YZ magneto-thermopower, if the field-dependent part of the signal originates entirely within the TI layer we would expect the voltage signal in the $Bi_2Se_3$/CoFeB bilayer to be reduced relative to the individual $Bi_2Se_3$ layer by shunting, i.e., $\frac{d}{\chi l \nabla T}$ should be similar to the two samples, using for the shunting parameter that $\chi \approx 1$ for the single layer and $\chi = \rho_{FM} t_{SS} / (\rho_{FM} t_{SS} + \rho_{SS} t_{FM}) \approx 0.16$ using symbols as defined in the main text. We find reasonable agreement with this expectation, with $\frac{d}{\chi l \nabla T}$ equal to $10.7 \times 10^{-3}$ $\frac{\mu V}{T^2 K}$ for the bilayer and $9.1 \times 10^{-3}$ $\frac{\mu V}{T^2 K}$ for the individual $Bi_2Se_3$.

Figure S6 shows a comparison between the $Bi_2Se_3$/CoFeB/$TaO_x$ and $Bi_2Se_3$/$TaO_x$ samples for the component of the $\cos^2 \theta$ magnetoconductance that depends quadratically on the magnetic field. We plot the change in conductance, rather than resistance, to allow a direct comparison between the two types of samples without needing to correct for the shunting factor. If the portion of the magnetoresistance corresponding to this signal originiated entirely in the TI layer, we should expect the two curves in Fig. S6 to be nearly identical. Instead, they differ in scale by a factor of about 3. This is surprising to us given that the corresponding quadratic-in-field

component of the magneto-thermopower is more similar between the samples. We speculate that the quadratic-in-field part of the magnetoconductance must be more sensitive to the existence of the TI's interface with the CoFeB layer, possibly due to either charge transfer or spin scattering.

# VI. Mott relations for magneto-thermopower and thermally generated spin currents

In metals and semimetals the flows of electrons in response to applied electric fields and thermal gradients are connected via Mott relations. For example, assuming linear response, the Seebeck coefficient ($S_{xx}$) is related to the electrical conductivity ($\sigma_{xx}$) (*22, 43*):

$$S_{xx} = \frac{-1}{eT\sigma_{xx}} \int -\frac{\partial f}{\partial E} \sigma_{xx}(E - E_f)dE \tag{15}$$

where $T$ is the temperature, $f$ is the Fermi-Dirac distribution, and $E_f$ denotes the energy at the Fermi level. If we assume that only the states near the Fermi level contribute to thermal transport, i.e., for degenerate statistics, the Seebeck coefficient can be simplified as

$$S_{xx} = -\frac{\pi^2 k_B^2 T}{3\sigma_{xx} e} \frac{\partial \sigma_{xx}}{\partial E}. \tag{16}$$

This equation can be used to obtain a relation between magneto-thermopower and magneto-resistance assuming $\sigma_{xx} = \sigma_0 + \sigma_1 B^2 + O(B^4)$, where $\sigma_1$ denotes the magento-resistance, and similarly $S_{xx} = S_{xx}^0 + S_{xx}^1 B^2 + O(B^4)$. The magneto-thermopower coefficient ($S_{xx}^1$) is related to conductivity as

$$S_{xx}^1 = -\frac{\pi^2 k_B^2 T}{3\sigma_0 e} \left( \frac{\partial \sigma_1}{\partial E} - \frac{\sigma_1}{\sigma_0} \frac{\partial \sigma_0}{\partial E} \right). \tag{17}$$

For $Bi_2Se_3$, both $\sigma_1$ (*44*) and $\frac{\partial \sigma_0}{\partial E}$ are large (both semimetallic surface states and semiconducting bulk show large change in conductivity with change in Fermi level), and hence a strong

magneto-thermopower is expected and observed. A similar connection can also be derived between the spin Nernst effect and the spin Hall effect. The spin current generated by spin Nernst effect can be written (*22*)

$$\frac{J_s}{\nabla T} = -\frac{1}{eT}\frac{\hbar}{2e}\int -\frac{\partial f}{\partial E}\sigma_{SH}(E-E_f)dE. \tag{18}$$

Similar to Seebeck effect, the thermally generated spin current can be simplified as

$$\frac{J_s}{\nabla T} = -\frac{\hbar}{2e}\frac{\pi^2 k_B^2 T}{3e}\frac{\partial \sigma_{SH}}{\partial E}. \tag{19}$$

Furthermore, from Eq. (16) and Eq. (19), along with Eq. (2) in the main text, we can obtain the relation between spin Hall conductivity and spin Nernst ratio

$$\theta_{SN} = -\frac{\frac{\partial \sigma_{SH}}{\partial E}}{\frac{\partial \sigma}{\partial E}} = -(\theta_{SH} + \frac{\frac{\partial \theta_{SH}}{\partial E}}{\frac{\partial \sigma}{\partial E}}). \tag{20}$$

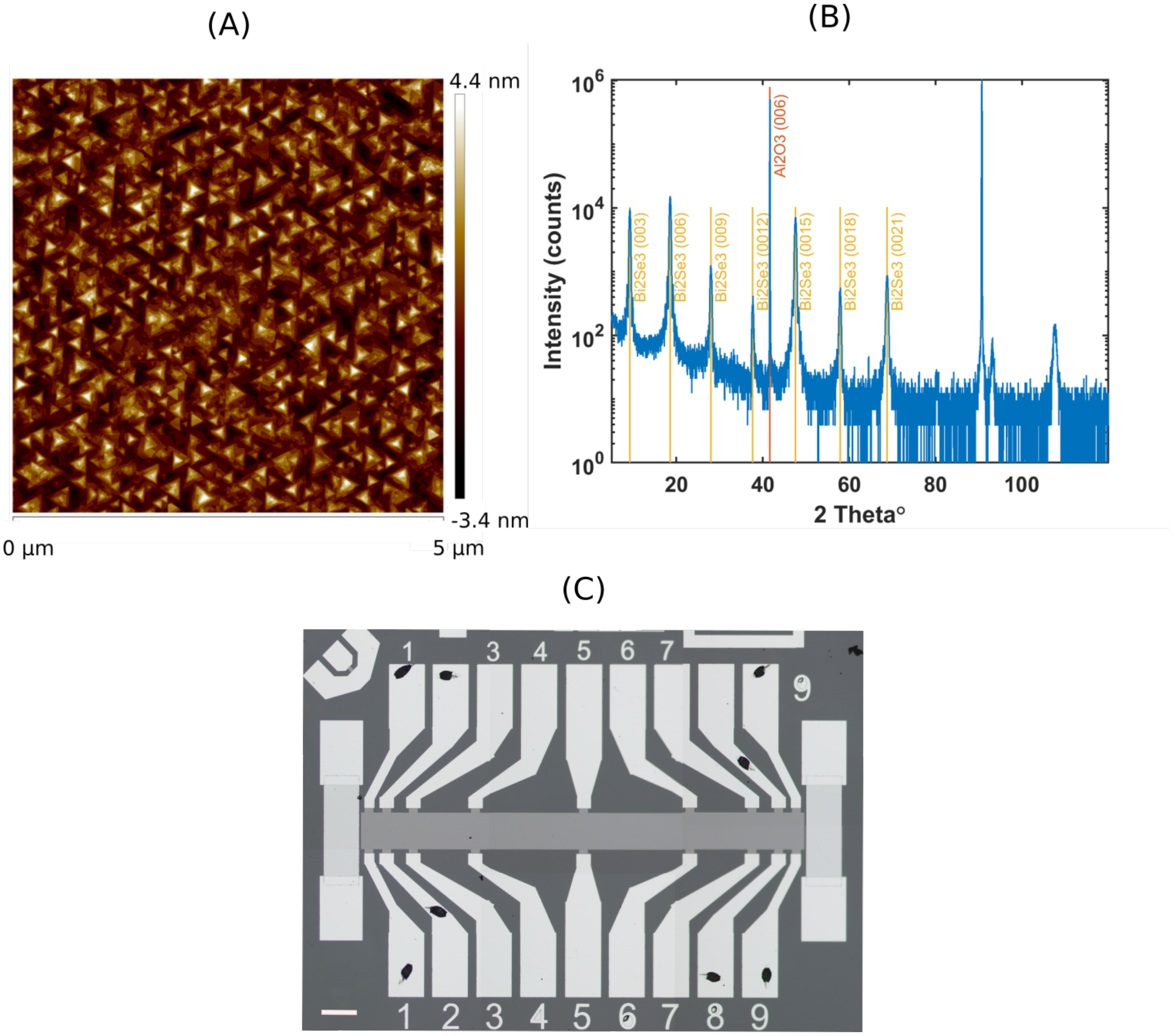


Figure S1: **Materials characterization of the $Bi_2Se_3$ layer (with no Se capping layer).** (A) Atomic force microscope image of the $Bi_2Se_3$ surface after growth, indicating a root-mean-square roughness of 1.13 nm. (B) X-ray reflectivity measurement of a $Bi_2Se_3$ sample indicating high-quality growth with sharp interfaces. (C) Microscope image of the device used for measuring spin Nernst magneto-thermopower. Scale bar is equivalent to 200 µm.

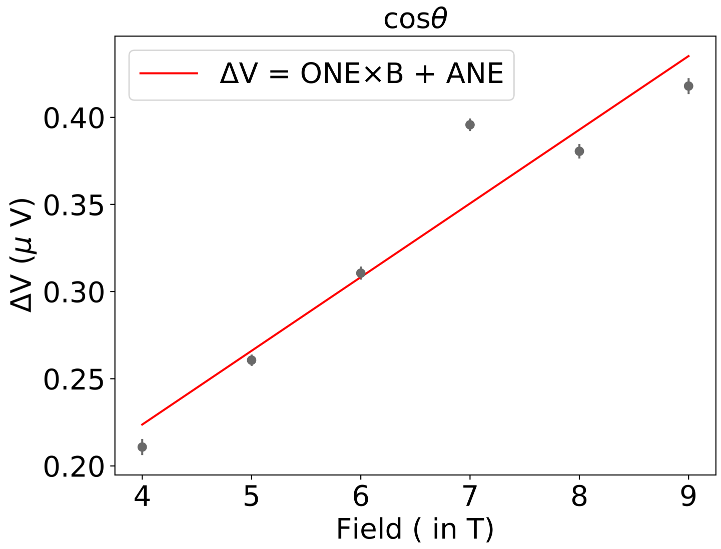


Figure S2: **Field dependence of the cosθ part of YZ magneto-thermopower.** Data in Fig. 2(B) with a fit to the form ONE $\times$ B + ANE with ONE = 0.042 $\pm$ 0.006 μV/T and ANE = 0.05 $\pm$ 0.04 μV. ONE and ANE refer to voltages arising from ordinary Nernst effect and anomalous Nernst effect, respectively.

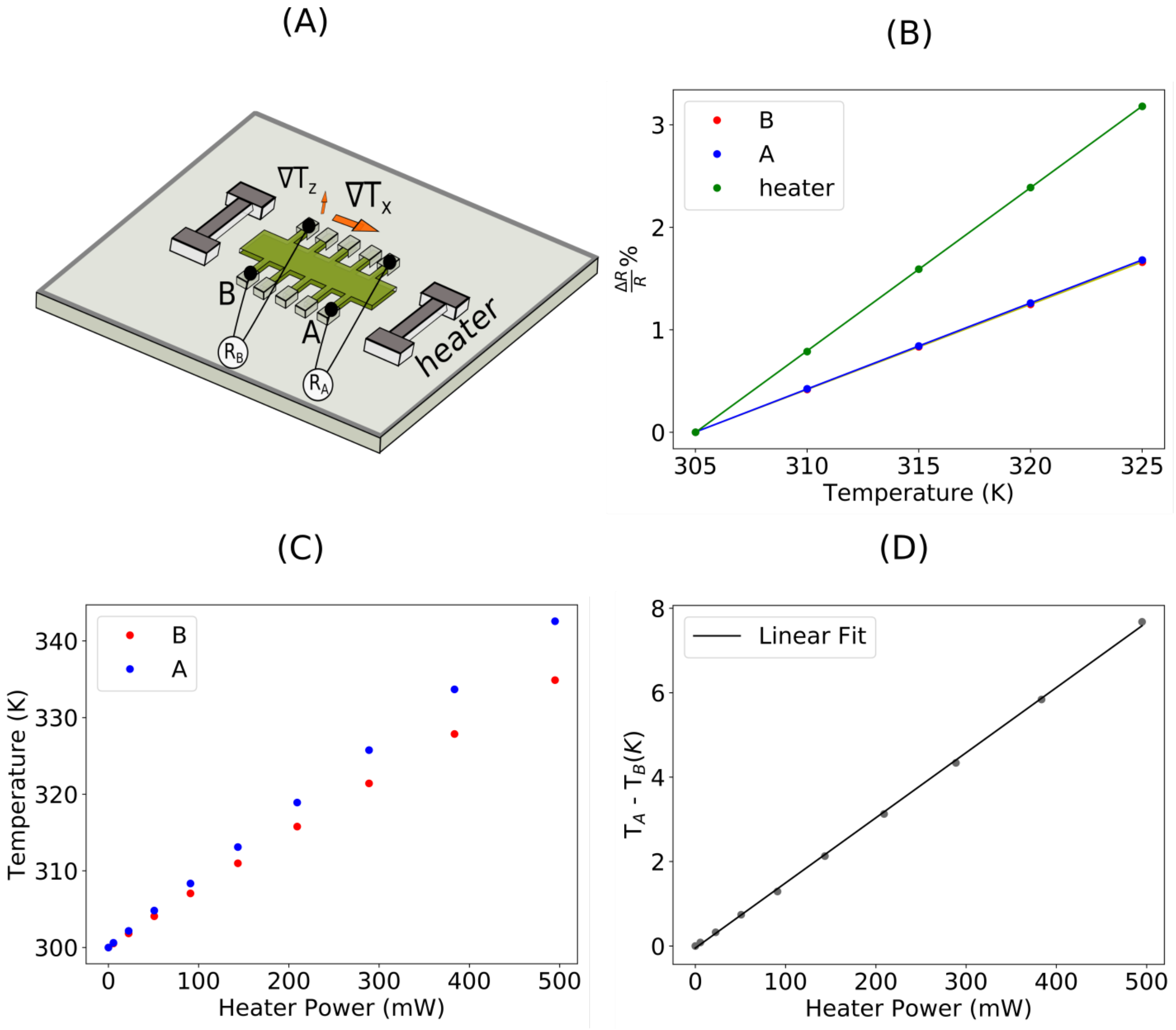


Figure S3: **Procedure for calibrating the temperature difference between the points on the sample used for the spin Nernst magneto-thermopower measurement.** (A) Sample schematic. (B) Calibration of change in resistance for contacts at points A and B and the heater as a function of sample temperature. (The data for points A and B closely overlap.) We fit the ratios $\frac{\Delta R}{R}$ to a linear function $100 \times \frac{\Delta R}{R} = \alpha(T - T_0)$ where $T_0$ is chosen to be 305 K. Linear fits yield $\alpha$ to be 0.083, 0.084, and 0.159 $K^{-1}$ for points A, B, and the heater respectively. (C) Change in temperature at the points A and B on the sample as the function of heater power. The temperature changes are determined from the measured changes in resistance, and calibrated using the data in (B). (D) Temperature difference between points A and B as a function of heater power calculated from the difference in (C).

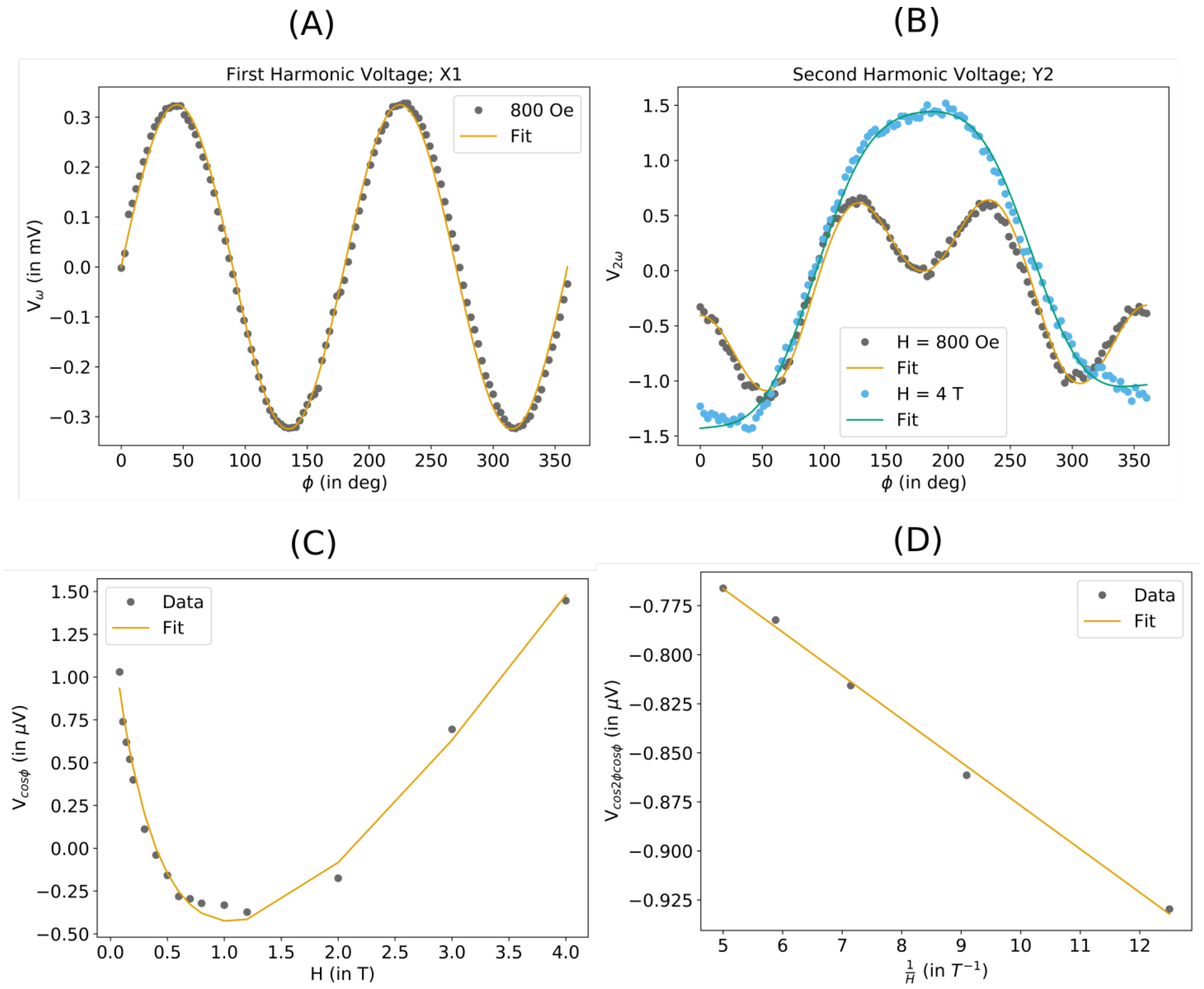


Figure S4: **Second harmonic Hall analysis of spin-orbit torques in a bilayer of $Bi_2Se_3$(8 nm)/CoFeB(2.6 nm).** (A) First harmonic Hall voltage at an external magnetic field of 800 Oe applied in plane as a function of the field angle. (B) Second Harmonic voltages at 800 Oe and 4 T as a function of the angle of the in-plane magentic field. (C) $\cos\phi$ component of the second harmonic Hall voltage versus the magnitude of the applied field. (D) $\cos 2\phi \cos\phi$ component of the second harmonic Hall voltage versus the inverse magnitude of the applied field.

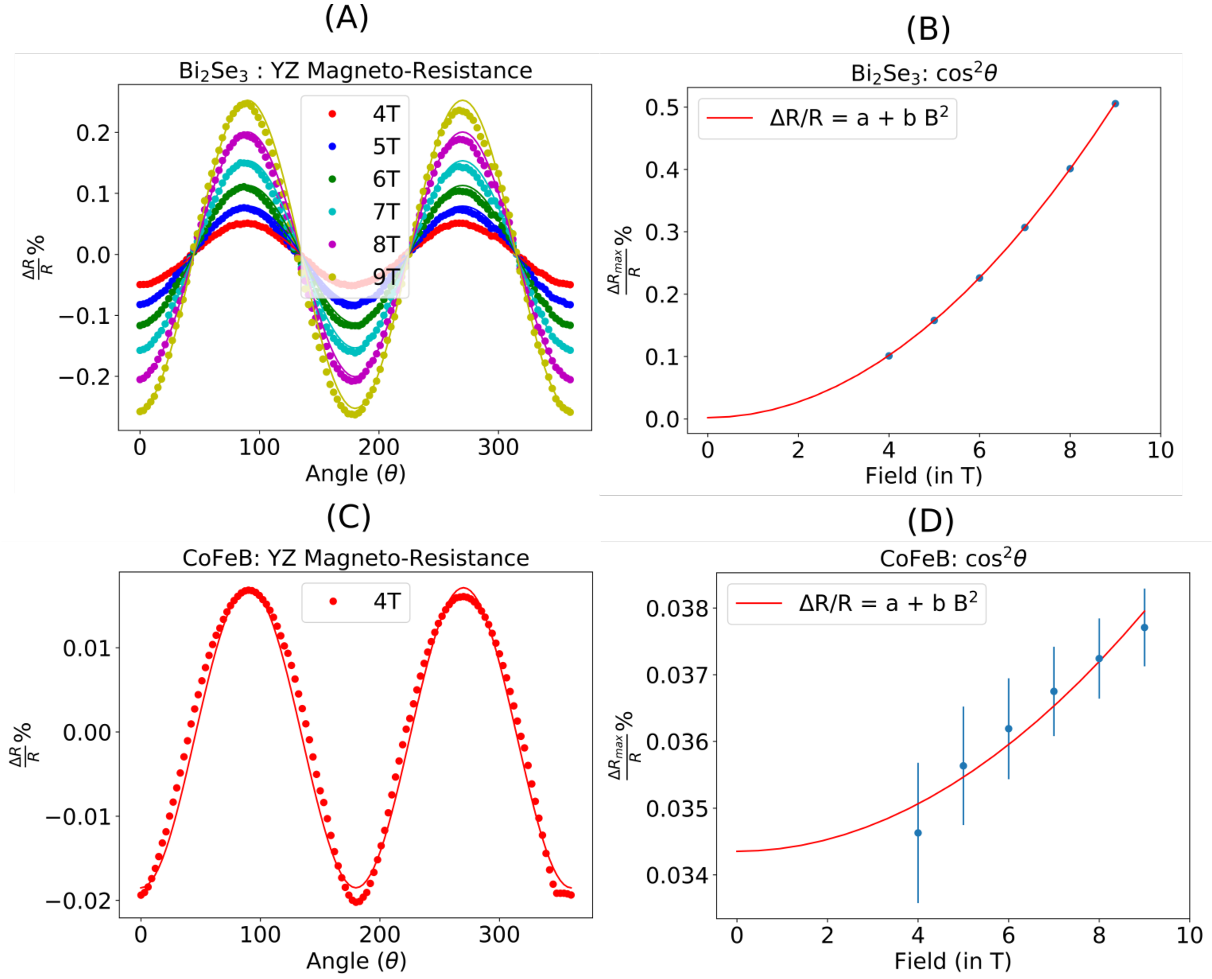


Figure S5: **YZ magnetoresistance of single layers of $Bi_2Se_3$ and CoFeB.** (A) Magnetoresistance percentage ratio ($\frac{\nabla R}{R} \times 100$) as a function of the magnetic field angle and magnitude for $Bi_2Se_3$ (8 nm) at room temperature, for magnetic field rotated in the YZ plane. (B) Amplitude of the YZ magnetoresistance for $Bi_2Se_3$ as a function of magnetic-field magnitude, with a fit to the form $100 \times (a + bB^2)$. The fit yields $a = 0.0020 \pm 0.0006$ and $b = 0.00622 \pm 0.00002$ $\mathrm{T}^{-2}$. (C) Magnetoresistance percentage ratio ($\frac{\Delta R}{R} \times 100$) as a function of the magnetic field angle and magnitude for CoFeB (5 nm) at room temperature, for magnetic field rotated in the YZ plane. (D) Amplitude of the YZ magnetoresistance for CoFeB as a function of magnetic-field magnitude, with a fit to the form $100 \times (a + bB^2)$. The fit yields $a = 0.034 \pm 0.003$ and $b = (4.4 \pm 0.6) \times 10^{-5}$ $\mathrm{T}^{-2}$.

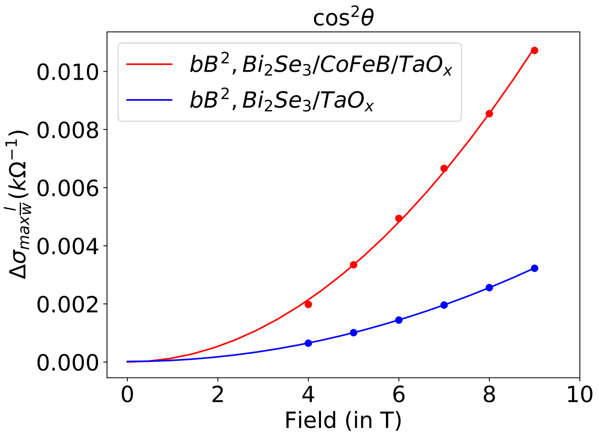


Figure S6: **Comparison of the scaled change in the quadratic-in-magnetic-field portion of the YZ magneto-conductance of $Bi_2Se_3$ with and without magnetic layer.** $\Delta\sigma_{max}\frac{l}{w}$ for $Bi_2Se_3/TaO_x$ and $Bi_2Se_3/CoFeB/TaO_x$, with fits to the form $bB^2$. (The field-independent part is omitted for clarity.) The fits yields $b = (3.970 \pm 0.008) \times 10^{-5} k\Omega^{-1}$ $T^{-2}$ and $b$ = $(13.4 \pm 0.2)$ $\times\ 10^{-5}$ $k\Omega^{-1}$ $T^{-2}$ respectively for $Bi_2Se_3/TaO_x$ and $Bi_2Se_3/CoFeB/TaO_x$.

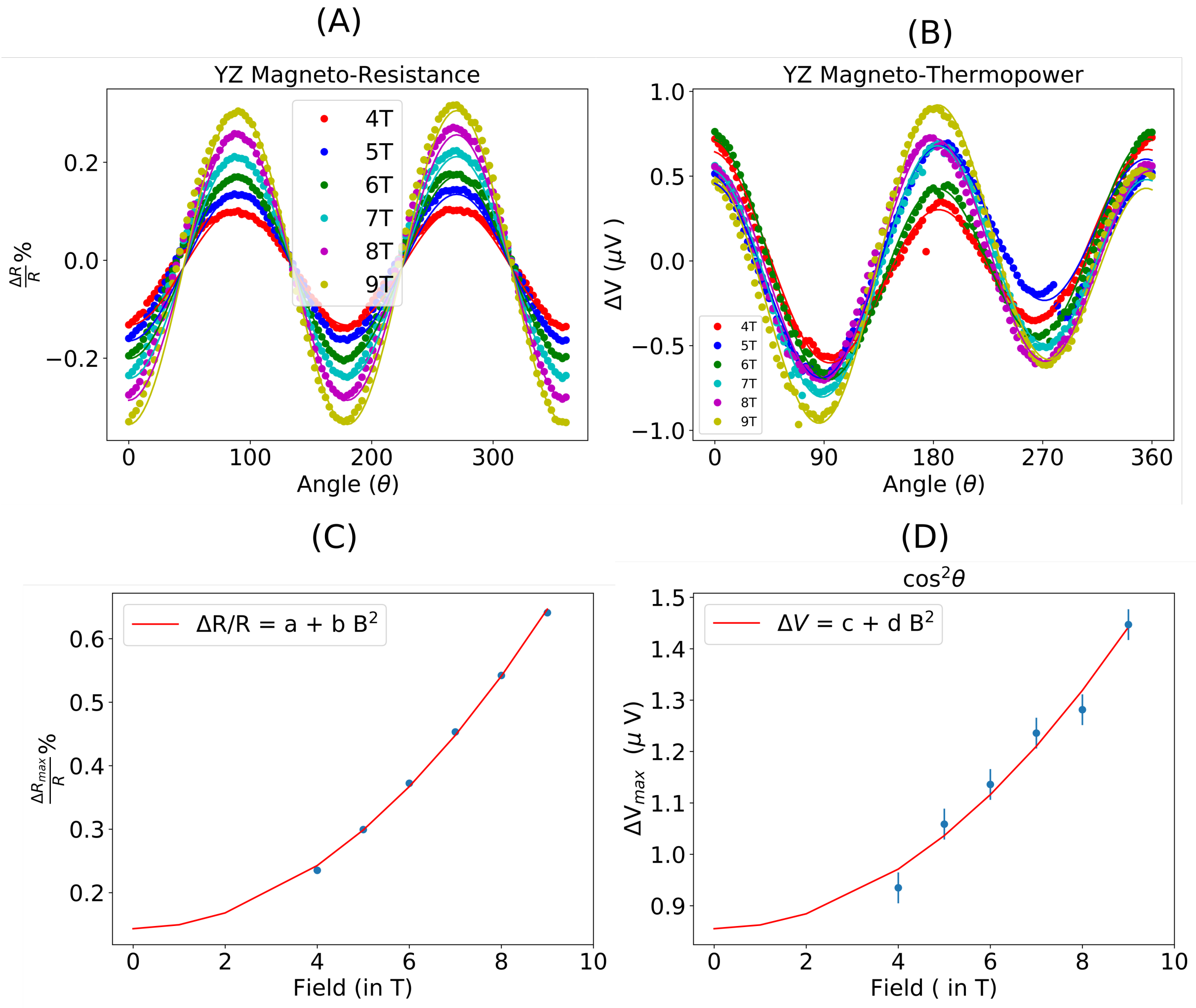


Figure S7: **Electrically and thermally generated spin currents in $Bi_2Se_3$ (8 nm)/CoFeB (6 nm)** (A) Magnetoresistance percentage ratio ($\frac{\nabla R}{R} \times 100$) as a function of the magnetic field angle and magnitude for bilayers of $Bi_2Se_3$ (8 nm)/CoFeB (6 nm) at room temperature, for magnetic field rotated in the YZ plane. The four-point device resistance $R$ = 1.967 kΩ. (B) YZ magneto-thermopower voltage as a function of the magnetic field angle and magnitude for the same sample. The heater power used for these sweeps was fixed at 513 mW (equivalent to a temperature drop of 8.5 ± 0.3 K along the $l$ = 1.8 mm length of the device.) (C) Amplitude of the YZ magnetoresistance as a function of magnetic-field magnitude, with a fit to the form $100 \times (a + bB^2)$. The fit yields $a = 0.143 \pm 0.05$ and $b = 0.0062 \pm 0.0001$ $T^{-2}$. (D) Field dependence of the $\cos^2\theta$ part of YZ magneto-thermopower with a fit to the form $c + dB^2$. The fit yields $c = 0.855 \pm 0.08$ μV and $d = 0.0072 \pm 0.0006$ μV/$T^2$. For this bilayer, we calculate the $S_{SNT}$ to be 101 ± 13 $\frac{nV}{K}$ consistent with the bilayer analyzed in the main text.

**REFERENCES AND NOTES**


1. G. E. W. Bauer, E. Saitoh, B. J. Van Wees, Spin caloritronics. *Nat. Mater.* **11**, 391–399 (2012).

2. S. R. Boona, R. C. Myers, J. P. Heremans, Spin caloritronics. *Energy Envron. Sci.* **7**, 885–910 (2014).

3. K.-I. Uchida, R. Iguchi, Spintronic thermal management. *J. Phys. Soc. Japan* **90**, 122001 (2021).

4. A. R. Mellnik, J. S. Lee, A. Richardella, J. L. Grab, P. J. Mintun, M. H. Fischer, A. Vaezi, A. Manchon, E.-A. Kim, N. Samarth, D. C. Ralph, Spin-transfer torque generated by a topological insulator. *Nature* **511**, 449–451 (2014).

5. Y. Fan, P. Upadhyaya, X. Kou, M. Lang, S. Takei, Z. Wang, J. Tang, L. He, L.-T. Chang, M. Montazeri, G. Yu, W. Jiang, T. Nie, R. N. Schwartz, Y. Tserkovnyak, K. L. Wang, Magnetization switching through giant spin–orbit torque in a magnetically doped topological insulator heterostructure. *Nat. Mater.* **13**, 699–704 (2014).

6. H. Wu, P. Zhang, P. Deng, Q. Lan, Q. Pan, S. A. Razavi, X. Che, L. Huang, B. Dai, K. Wong, X. Han, K. L. Wang, Room-temperature spin-orbit torque from topological surface states. *Phys. Rev. Lett.* **123**, 207205 (2019).

7. M. Jamali, J. S. Lee, J. S. Jeong, F. Mahfouzi, Y. Lv, Z. Zhao, B. K. Nikolić, K. A. Mkhoyan, N. Samarth, J.-P. Wang, Giant spin pumping and inverse spin Hall effect in the presence of surface and bulk spin-orbit coupling of topological insulator $Bi_2Se_3$. *Nano Lett.* **15**, 7126–7132 (2015).

8. H. Wang, J. Kally, J. S. Lee, T. Liu, H. Chang, D. R. Hickey, K. A. Mkhoyan, M. Wu, A. Richardella, N. Samarth, Surface-state-dominated spin-charge current conversion in topological-insulator–ferromagnetic-insulator heterostructures. *Phys. Rev. Lett.* **117**, 076601 (2016).

9. J. Han, A. Richardella, S. A. Siddiqui, J. Finley, N. Samarth, L. Liu, Room-temperature spin-orbit torque switching induced by a topological insulator. *Phys. Rev. Lett.* **119**, 077702 (2017).

10. M. Dc, R. Grassi, J.-Y. Chen, M. Jamali, D. R. Hickey, D. Zhang, Z. Zhao, H. Li, P. Quarterman, Y. Lv, M. Li, A. Manchon, K. A. Mkhoyan, T. Low, J.-P. Wang, Room-temperature high spin–orbit torque due to quantum confinement in sputtered $Bi_xSe_{(1-x)}$ films. *Nat. Mater.* **17**, 800–807 (2018).

11. N. H. D. Khang, Y. Ueda, P. N. Hai, A conductive topological insulator with large spin Hall effect for ultralow power spin–orbit torque switching. *Nat. Mater.* **17**, 808–813 (2018).

12. C. J. Vineis, A. Shakouri, A. Majumdar, M. G. Kanatzidis, Nanostructured thermoelectrics: Big efficiency gains from small features. *Adv. Mater.* **22**, 3970–3980 (2010).

13. Y. Hor, A. Richardella, P. Roushan, Y. Xia, J. Checkelsky, A. Yazdani, M. Hasan, N. Ong, R. Cava, *p*-type $Bi_2Se_3$ for topological insulator and low-temperature thermoelectric applications. *Phys. Rev. B* **79**, 195208 (2009).

14. M. Guo, Z. Wang, Y. Xu, H. Huang, Y. Zang, C. Liu, W. Duan, Z. Gan, S.-C. Zhang, K. He, X. Ma, Q. Xue, Y. Wang, Tuning thermoelectricity in a $Bi_2Se_3$ topological insulator via varied film thickness. *New J. Phys.* **18**, 015008 (2016).

15. N. Roschewsky, E. S. Walker, P. Gowtham, S. Muschinske, F. Hellman, S. R. Bank, S. Salahuddin, Spin-orbit torque and Nernst effect in Bi-Sb/Co heterostructures. *Phys. Rev. B* **99**, 195103 (2019).

16. J.-M. Kim, D.-J. Kim, C.-Y. Cheon, K.-W. Moon, C. Kim, P. Cao Van, J.-R. Jeong, C. Hwang, K.-J. Lee, B.-G. Park, Observation of thermal spin–orbit torque in W/CoFeB/MgO structures. *Nano Lett.* **20**, 7803–7810 (2020).

17. A. Bose, A. S. Shukla, S. Dutta, S. Bhuktare, H. Singh, A. A. Tulapurkar, Control of magnetization dynamics by spin-Nernst torque. *Phys. Rev. B* **98**, 184412 (2018).

18. H. Nakayama, M. Althammer, Y.-T. Chen, K.-I. Uchida, Y. Kajiwara, D. Kikuchi, T. Ohtani, S. Geprägs, M. Opel, S. Takahashi, R. Gross, G. E. W. Bauer, S. T. B. Goennenwein, E. Saitoh, Spin Hall magnetoresistance induced by a nonequilibrium proximity effect. *Phys. Rev. Lett.* **110**, 206601 (2013).

19. L. Liu, C.-F. Pai, Y. Li, H. W. Tseng, D. C. Ralph, R. A. Buhrman, Spin-torque switching with the giant spin Hall effect of tantalum. *Science* **336**, 555–558 (2012).

20. Y.-T. Chen, S. Takahashi, H. Nakayama, M. Althammer, S. T. B. Goennenwein, E. Saitoh, G. E. W. Bauer, Theory of spin Hall magnetoresistance. *Phys. Rev. B* **87**, 144411 (2013).

21. J. Kim, P. Sheng, S. Takahashi, S. Mitani, M. Hayashi, Spin Hall magnetoresistance in metallic bilayers. *Phys. Rev. Lett.* **116**, 097201 (2016).

22. S. Meyer, Y.-T. Chen, S. Wimmer, M. Althammer, T. Wimmer, R. Schlitz, S. Geprägs, H. Huebl, D. Ködderitzsch, H. Ebert, G. E. W. Bauer, R. Gross, S. T. B. Goennenwein , Observation of the spin Nernst effect. *Nat. Mater.* **16**, 977–981 (2017).

23. A. Bose, A. A. Tulapurkar, Recent advances in the spin Nernst effect. *J. Magn. Magn. Mater.* **491**, 165526 (2019).

24. P. Sheng, Y. Sakuraba, Y.-C. Lau, S. Takahashi, S. Mitani, M. Hayashi, The spin Nernst effect in tungsten. *Sci. Adv.* **3**, e1701503 (2017).

25. D.-J. Kim, C.-Y. Jeon, J.-G. Choi, J. W. Lee, S. Surabhi, J.-R. Jeong, K.-J. Lee, B.-G. Park, Observation of transverse spin Nernst magnetoresistance induced by thermal spin current in ferromagnet/non-magnet bilayers. *Nat. Commun.* **8**, 1400 (2017).

26. K. Tauber, M. Gradhand, D. V. Fedorov, I. Mertig, Extrinsic spin nernst effect from first principles. *Phys. Rev. Lett.* **109**, 026601 (2012).

27. S. Wimmer, D. Ködderitzsch, K. Chadova, H. Ebert, First-principles linear response description of the spin nernst effect. *Phys. Rev. B* **88**, 201108 (2013).

28. Y. Zhang, K. He, C.-Z. Chang, C.-L. Song, L.-L. Wang, X. Chen, J.-F. Jia, Z. Fang, X. Dai, W.-Y. Shan, S.-Q. Shen, Q. Niu, X.-L. Qi, S.-C. Zhang, X.-C. Ma, Q.-K. Xue, Crossover of the three-dimensional topological insulator $Bi_2Se_3$ to the two-dimensional limit. *Nat. Phys.* **6**, 584–588 (2010).

29. M. Neupane, A. Richardella, J. Sánchez-Barriga, S. Xu, N. Alidoust, I. Belopolski, C. Liu, G. Bian, D. Zhang, D. Marchenko, A. Varykhalov, O. Rader, M. Leandersson, T. Balasubramanian, T.-R. Chang, H.-T. Jeng, S. Basak, H. Lin, A. Bansil, N. Samarth, M. Z. Hasan, Observation of quantum-tunnelling-modulated spin texture in ultrathin topological insulator $Bi_2Se_3$ films. *Nat. Commun.* **5**, 3841 (2014).

30. A. Bose, R. Jain, J. J. Bauer, R. A. Buhrman, C. A. Ross, D. C. Ralph, Origin of transverse voltages generated by thermal gradients and electric fields in ferrimagnetic-insulator/heavy-metal bilayers. *Phys. Rev. B* **105**, L100408 (2022).

31. M. Kockert, R. Mitdank, A. Zykov, S. Kowarik, S. Fischer, Absolute Seebeck coefficient of thin platinum films. *J. Appl. Phys.* **126**, 105106 (2019).

32. M. Hayashi, J. Kim, M. Yamanouchi, H. Ohno, Quantitative characterization of the spin-orbit torque using harmonic Hall voltage measurements. *Phys. Rev. B* **89**, 144425 (2014).

33. Z. Jiang, C.-Z. Chang, M. R. Masir, C. Tang, Y. Xu, J. S. Moodera, A. H. MacDonald, J. Shi, Enhanced spin Seebeck effect signal due to spin-momentum locked topological surface states. *Nat. Commun.* **7**, 11458 (2016).

34. M. Mogi, K. Yasuda, R. Fujimura, R. Yoshimi, N. Ogawa, A. Tsukazaki, M. Kawamura, K. S. Takahashi, M. Kawasaki, Y. Tokura, Current-induced switching of proximity-induced ferromagnetic surface states in a topological insulator. *Nat. Commun.* **12**, 1404 (2021).

35. H. Wang, J. Kally, C. Şahin, T. Liu, W. Yanez, E. J. Kamp, A. Richardella, M. Wu, M. E. Flatté, N. Samarth, Fermi level dependent spin pumping from a magnetic insulator into a topological insulator. *Phys. Rev. Res.* **1**, 012014 (2019).

36. J. Zhang, J. P. Velev, X. Dang, E. Y. Tsymbal, Band structure and spin texture of $Bi_2Se_3$ 3*d* ferromagnetic metal interface. *Phys. Rev. B* **94**, 014435 (2016).

37. Y.-T. Hsu, K. Park, E.-A. Kim, Hybridization-induced interface states in a topological-insulator–ferromagnetic-metal heterostructure. *Phys. Rev. B* **96**, 235433 (2017).

38. R. Jain, M. Stanley, A. Bose, A. R. Richardella, X. Zhang, T. Pillsbury, D. A. Muller, N. Samarth, D. C. Ralph, Thermally-generated spin current in the topological insulator $Bi_2Se_3$. arXiv:2210.05636 [cond-mat.mes-hall] (11 October 2022).

39. D. MacNeill, G. M. Stiehl, M. H. Guimaraes, N. D. Reynolds, R. A. Buhrman, D. C. Ralph, Thickness dependence of spin-orbit torques generated by $WTe_2$. *Phys. Rev. B* **96**, 054450 (2017).

40. K. Yasuda, A. Tsukazaki, R. Yoshimi, K. Kondou, K. Takahashi, Y. Otani, M. Kawasaki, Y. Tokura, Current-nonlinear hall effect and spin-orbit torque magnetization switching in a magnetic topological insulator. *Phys. Rev. Lett.* **119**, 137204 (2017).

41. Y. Wang, Y. Wang, T. Wang, X. Wang, Y. Ou, Y. Ji, M. F. Doty, S. A. Law, J. Q. Xiao, One-dimensional antilocalization of electrons from spin disorder probed by nonlinear hall effect. *Phys. Rev. B* **102**, 125430 (2020).

42. T. Seki, Y.-C. Lau, S. Iihama, K. Takanashi, Spin-orbit torque in a Ni-Fe single layer. *Phys. Rev. B* **104**, 094430 (2021).

43. M. Jonson, G. Mahan, Mott's formula for the thermopower and the wiedemann-franz law. *Phys. Rev. B* **21**, 4223(1980).

44. S. Wiedmann, A. Jost, B. Fauqué, J. Van Dijk, M. Meijer, T. Khouri, S. Pezzini, S. Grauer, S. Schreyeck, C. Brüne, H. Buhmann, L. W. Molenkamp, N. E. Hussey, Anisotropic and strong negative magnetoresistance in the three-dimensional topological insulator $Bi_2Se_3$. *Phys. Rev. B* **94**, 081302 (2016).
